# Improving risk prediction via predicting coronary plaque volume

**DOI:** 10.1016/j.ajpc.2026.101554

**Published:** 2026-03-13

**Authors:** Rishi Rikhi, Michael J. Blaha, Ron Blankstein

**Affiliations:** a Division of Cardiovascular Medicine, Department of Medicine, Brigham and Women’s Hospital, Harvard Medical School, Boston, MA, USA; b Johns Hopkins Ciccarone Center for the Prevention of Cardiovascular Disease, Baltimore, Maryland, USA

**Keywords:** Coronary atherosclerosis, Genomics, Prevention, Lipids

In primary prevention, risk assessment with conventional risk scores is the foundation for guiding therapies to delay the onset and progression of atherosclerosis, as well as reduce atherosclerotic cardiovascular disease (ASCVD) events [1]. Despite many advances ranging from genetics to novel biomarkers, a need exists to improve risk assessment, especially among individuals with borderline or intermediate risk [2].

Coronary artery calcium (CAC) is commonly used to refine risk when there is uncertainty regarding statin initiation and serves as a direct measure of plaque, capturing long term exposure to both known and unknown risk factors [1]. In fact, when combined with conventional risk scores, CAC significantly improves ASCVD risk prediction, even among low-risk individuals [3]. Recent advancements in omics research have allowed for the development of risk scores that capture genetic and metabolic risk, which have been shown to modestly improve ASCVD risk prediction [4,5]. However, data are limited regarding whether genomic and lipidomic profiling provide incremental value beyond coronary plaque volume or improve prediction of plaque burden.

The extent and severity of coronary plaque can be assessed by coronary computed tomographic angiography (CCTA), and recent advances in artificial intelligence techniques have enabled quantitative coronary plaque analysis [6]. Importantly, the amount of plaque detected by CAC or CCTA has powerful prognostic implications, as individuals with higher plaque burden have greater ASCVD risk, irrespective of whether obstructive or non-obstructive coronary artery disease (CAD) is present [7]. Given this information, it seems potentially compelling that combining CAC, genetic, and lipidomic information could help improve risk assessment and identify individuals where further imaging or treatment may be indicated.

In this issue of *American Journal of Preventive Cardiology*, Soh et al. investigate the predictive value of a Lipidomic Risk Score (LRS), Polygenic Risk Score (PRS), and CAC in identifying coronary artery plaque volume when added to the Multi-Ethnic Study of Atherosclerosis (MESA) 10-year cardiovascular risk score [8]. Here, the LRS included 705 lipid species that was validated in an Australian cohort and the PRS was derived from the UK Biobank cohort [8]. The cross-sectional study included a development cohort (mean age 58.5 years, 43.1 % female) of 232 participants from GenoCAD and an external validation cohort (mean age 61.7 years, 28.1 % female) of 199 participants from the Early Detection of CAD by Polygenic Risk Scoring (EDCAD-PMS) [8]. All participants were statin-naïve with a family history of CAD [8].

The authors found a weak correlation between LRS and PRS with total plaque volume, whereas CAC showed a moderate correlation with both total plaque volume and non-calcified plaque volume [8]. Compared to the 10-year MESA cardiovascular risk score, the addition of PRS and LRS to the model increased the AUC for high coronary total plaque volume, defined as >48mm[3], modestly from 0.498 to 0.552 in the validation cohort [8]. In contrast, the addition of CAC to the 10-year MESA cardiovascular risk score increased the AUC substantially from 0.498 to 0.823 [8]. Overall, the highest predictive performance for high coronary total plaque volume was seen when PRS, LRS, and CAC were all added to the 10-year MESA cardiovascular risk score (AUC=0.913) [8]. A similar improvement in model fit was found in a sensitivity analysis building upon the more recently developed Predicting Risk of Cardiovascular Disease EVENTs (PREVENT) risk score [8].

Certain limitations exist in this study [8]. All participants had a family history of CAD [8]. Thus, the overall population had elevated genetic risk, which may have limited the predictive value of PRS [8]. Additionally, CCTA was only performed in those with a CAC>0 and <400, limiting generalizability to individuals without CAC, and generally lower-risk individuals [8]. In primary prevention, individuals without CAC and a 10-year ASCVD risk <20 % may have ASCVD event rates that are below the level of statin initiation [9]. Therefore, the 2018 American Heart Association (AHA)/American College of Cardiology (ACC) Blood Cholesterol Guideline provide an option to delay or withhold statin therapy in intermediate risk individuals with a CAC of zero, if the decision for statin initiation is uncertain [1]. However, this recommendation does not apply to those with diabetes, current smoking, and most relevant to the current study, strong family history of premature ASCVD, as a CAC of zero does not sufficiently lower risk in these populations [1]. In the absence of CAC, PRS and LRS could have a potential role in further refining risk and identifying individuals with non-calcified plaque – a marker of higher long term risk even when CAC is zero – who could benefit from more aggressive primary prevention efforts. In addition, PRS and LRS could conceivably identify individuals without any plaque who have a higher risk of developing incident plaque. However, these concepts require testing in large cohorts of individuals with sufficiently long follow-up to observe plaque progression.

The authors should be congratulated for their novel and robust multi-omics approach toward coronary artery plaque volume prediction [8]. While conventional risk scores are fundamental for assessing risk in primary prevention, they may fail to capture individuals with atherosclerotic burden who are defined at low risk. For instance, in the current study, 54 % of individuals with a low 10-year MESA cardiovascular risk score (<5 %) had high coronary plaque volume [8]. However, the percentage of individuals with high total coronary plaque volume reduced to 25 % in participants who also had low PRS, LRS and CAC [8]. These data demonstrate the potential value of using omics and imaging in refining prediction of atherosclerotic disease burden.

This study highlights the distinct biological processes that are captured by conventional risk scores, PRS, LRS, and CAC [8]. Conventional risk scores assess exposure to risk factors, but may not always capture all relevant risk factors, or exposure to risk factors over time [10]. PRS measures inherited risk from genetic variants, while LRS estimates metabolic risk from the plasma lipidome [2,4]. On the other hand, CAC is a direct visual assessment of cumulative plaque burden [1]. It is thus not surprising that once CAC is present, it provides the most robust prognostic value, as was the case in the present study where the additional value of PRS and LRS was modest. These findings are in keeping with those of Khan et al., who found CAC to improve risk prediction for coronary heart disease when added to the pooled cohort equations, while PRS did not significantly improve risk prediction [2]. However, in another analysis by Edmin et al. from a much younger cohort, the Coronary Artery Risk development in Young Adults study, there was significantly greater risk among individuals without CAC in the highest PRS quintile compared to lowest PRS quntile [5]. Collectively, these findings highlight the unique biological processes captured by omics and CAC, and suggest PRS may be of possible value in younger individuals without CAC.

The field of prevention has seen rapid advancements in cardiac imaging, conventional risk scores, and multi-omics. Currently, CCTA is primarily used for assessing intermediate and high-risk individuals presenting with chest pain [11]. However, with further research, reduced costs, greater availability, and lower radiation protocols, it is conceivable that CCTA with quantitative plaque assessment may have a greater role in asymptomatic patients to guide prevention strategies [12]. While conventional risk scores are important tools for defining statin eligibility, PRS, LRS, and CAC may help identify, quantify, and contextualize plaque burden phenotypes to refine risk and identify individuals most likely to benefit from more aggressive treatment.

## CRediT authorship contribution statement

**Rishi Rikhi:** Writing – review & editing, Writing – original draft. **Michael J. Blaha:** Writing – review & editing, Writing – original draft. **Ron Blankstein:** Writing – review & editing, Writing – original draft.

## Declaration of competing interest

Dr. Blankstein reports consulting and research support from Amgen Inc, Novartis Inc, Nanox AI, Heartflow Inc, and Caristo Inc. The remaining authors declare that they have no known competing financial interests or personal relationships that could have appeared to influence the work reported in this paper.
